# RP11-552D4.1: a novel m6a-related LncRNA associated with immune status in glioblastoma

**DOI:** 10.18632/aging.204177

**Published:** 2022-07-18

**Authors:** Ping Zheng, Xiaoxue Zhang, Dabin Ren, Yisong Zhang

**Affiliations:** 1 Department of Neurosurgery, Shanghai Pudong New Area People’s Hospital, Shanghai, China; 2 Key Molecular Lab, Shanghai Pudong New Area People’s Hospital, Shanghai, China

**Keywords:** glioblastoma (GBM), TCGA, m6A, m6A-related lncRNA, RP11-552D4.1

## Abstract

Glioblastoma (GBM) is the most malignant form of brain cancer in the world. Nevertheless, the survival rate of patients with GBM is extremely low. N6-methyladenosine (m6A) and long noncoding RNAs (lncRNAs) conduct important biological functions in patients’ survival status and the immunotherapeutic response. Here, m6A-related lncRNAs were identified by a co-expression method. Univariate and multivariate Cox regression together with LASSO were applied to establish the risk model. Kaplan-Meier and ROC analysis were applied to evaluate the prediction power of this risk model. Finally, the related immune profiling and chemical sensitivity targets were also investigated. The risk model holding three m6A-related lncRNAs was confirmed as an independent predictor for the prognosis. Furthermore, we found the risk model based on m6A-related lncRNAs is associated with the immune status, immunosuppressive biomarkers, and chemo-sensitivity in GBM patients. The RP11-552D4.1 is found to facilitate neuronal proliferation. This risk model consisted of m6A-related lncRNAs may be available for the clinical interventions in GBM patients.

## INTRODUCTION

Glioma is one of the common malignant brain tumors, which accounts for 30% of adult brain cancer, and its incidence rate is rising every year [1, 2]. The most malignant pathological type is glioblastoma (GBM), which accounts for about 70–80% of adult glioma [2]. Compared with low-grade glioma (LGG) and astrocytoma, GBM demonstrates a very poor outcome and is more likely to metastasize [3]. When GBM appears to metastasize, its outcome is less favorable [4]. The curative treatment of early GBM is total resection. Nevertheless, one-third of patients are under a recurrence after brain surgery [5], even treated together with gene therapy and immune therapy, and the overall survival of GBM patients is still very shorter [6].

Several lncRNAs in the brain are specifically related to neuronal and synaptic function [7]. LncRNAs constitute a large class of post-transcriptional regulators, some of which can act as ceRNAs to inhibit microRNAs (miRNAs) in the brain [8, 9]. For instance, lnc -NKILA acts as a miR-195 sponge, leading to increased expression of miR-195 target gene *NLRX1*, particularly in neocortical and hippocampal neurons [10].

Epigenetic modification in GBM plays a key role in tumor initiation and progression [11, 12]. M6A is a dominant RNA modification on both coding and non-coding RNAs. A series of studies have demonstrated that m6A methylation has a critical effect in the tumor initiation and progression [13–15]. M6A-related genes can be divided into writers, erasers and readers [16, 17]. We previously reported that lncRNA-ATB may participate in tumorigenesis through targeting NF-κB and P38/MAPK [18]; On the other hand, lncRNA-ATB could also activate the astrocyte via miR-204-3p [19] and miR-200a [20] to promote the progression of glioma. However, lncRNA MEG3 can be regulated by DNA methylation and further inhibit glioma growth [21]. The double-faced role of lncRNAs in glioma might be due to its m6A modification style. Few studies have investigated the combination of m6A related lncRNAs in cancers, especially in GBM. Therefore, we constructed a GBM risk model to study the prognostic utility of m6A related genes in GBM.

## MATERIALS AND METHODS

### Data acquisition

**Figure 1:**
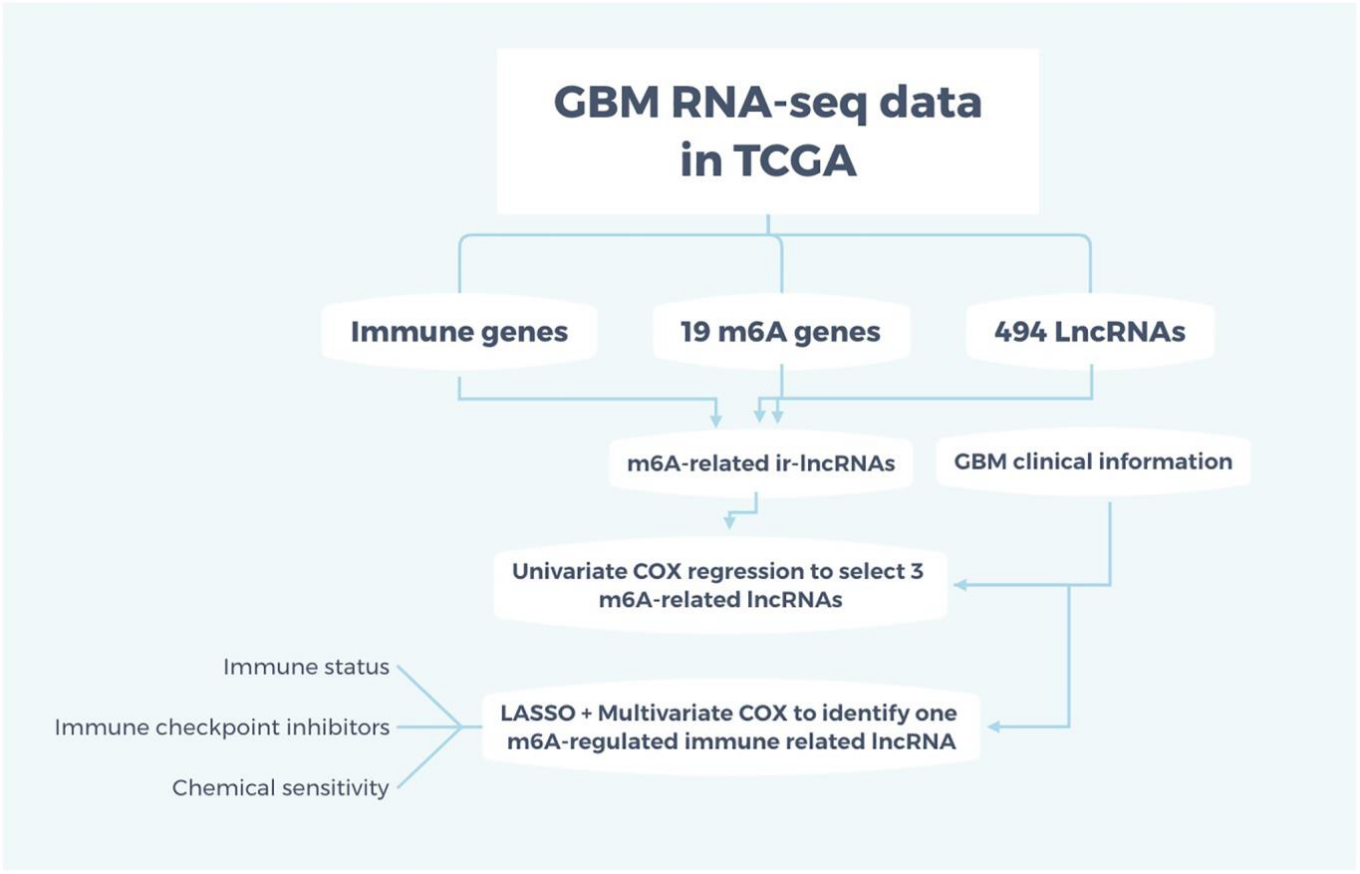
**Flow chart of the study.** The RNA-seq data for GBM were obtained from TCGA and m6A-related immune lncRNAs were intersected among immune genes, 19 m6A genes, and 494 differently-expressed lncRNAs. Adding the clinical information, univariate and multivariate COX regression and LASSO were applied to identify one m6A-regulated ir-lncRNA which is correlated with the survival status of GBM patients. Then, the ir-lncRNAs were analyzed for immune status, immune checkpoint inhibitors, and chemical sensitivity for GBM.

GBM data was available from the Cancer Genome Atlas (TCGA) on Oct 01, 2020, which consists of clinical data (survival status, age, sex, race), mutation files of the SNP, and gene expression profiles. Clinical information was available on 169 TCGA GBM samples and five control samples. Chinese Glioma Genome Atlas (CGGA) data are used for verification of the interested genes. Clinical information of GBM patients was downloaded from the TCGA website as well. Only the clinical information of patients with confirmed GBM pathology was included for final bioinformatic analysis. The candidate m6A gene sets were searched from “c2.cp.kegg. v7.0. symbols” of Gene Set Enrichment Analysis (GSEA). The correlation between lncRNAs and m6A genes was analyzed with co-expression methods. The inclusion criteria of m6A-related lncRNAs were based on Pearson coefficient more than 0.4 and *p*-value less than 0.001. The “Limma” R package was used to identify the m6A-related lncRNAs, and logFC >1 and false discovery rate (FDR) <0.05 was used to identify differently- expressed m6A-related lncRNA (DEmlncRNA). The flow chart for the current study is briefly summarized in Figure 1.

### Survival status correlation

We only included patients with GBM holding effective survival data from 2 days to 3881 days. Both univariate and multiple Cox model was used to assess the relationship between m6A-related lncRNAs and the survival status of GBM patient (*P* < 0.05). Risk score = Coefficient of mRNA × lncRNAs. The least absolute shrinkage and selection operator (LASSO) method is further applied to identify the exact coefficient of each DEmlncRNA based on the survival status. R packages “survival” and “survminer” were used to compare the survival status between the high-and low-risk group.

### Prognostic signature construction

The survivalROC package was used to draw the Receiver Operating Characteristic (ROC) curve, in order to evaluate the prediction ability of the risk model. By integrating clinical features into Cox models, a prognostic signature was identified as dependent or independent factors. In addition, we drew the one-, two- and three-year ROC curves and obtained the highest area under curve (AUC) value.

### Tumor-infiltrating cells (TILs) in GBM

We next investigated the association between risk group and TILs, with different immune infiltration analysis algorithm. Wilcoxon signed-rank test was used to compare the number of TILs between high- and low-risk groups. The correlation between riskScores and TILs was investigated by the Spearman method. A lollipop plot shows the correlation coefficient.

### Expression of immune-checkpoint inhibitor (ICI) markers

The correlation between riskScores and the ICI markers was investigated by Wilcoxon signed-rank test and further visualized with ggstatsplot package.

### The association between the risk model and the clinical therapy

In order to evaluate the clinical value of the risk model in GBM, the half maximal inhibitory concentration (IC50) of commonly used chemotherapy drugs for GBM patients was investigated. Most medicines were evaluated with pRRophetic package. Wilcoxon signed-rank test was carried out to compare the different IC50 between the high- and low-risk scores.

### Reagents and antibodies

Mouse monoclonal to MAP2 (ab11267, Abcam) and rabbit monoclonal to β-tubulin (ab201831, Abcam) were utilized to evaluate the cell proliferation for IFC.

### Primary cortical neuronal cultures and plasmids treated IFC

Primary culture of cortical neurons was previously reported [22]. Plasmid-mediated lncRNA overexpression and knockdown vector were obtained from Yazai biotech (Shanghai, China), siRNAs targeting RP11-552D4.1 were bought from Thermo Fisher Scientific (n505669, Shanghai, China) and control plasmid was ordered from GeneCopoeia (Rockville, MD, USA). The immunofluorescence (IFC) method was previously reported as well. In this study, we probed the cultured neurons with MAP2 and β-tubulin overnight and the secondary antibody was HRP.

### CCK-8 assay

Each group of cells was adjusted at 1,000 cells per well. 10 mL of CCK-8 solution (Beyotime Biotechnology, Shanghai, China) was added to the cell dish after 24 hours, and a blank control has only CCK-8 solution. Absorbance (OD) value of each well was read for each well at 450 nm and tested every 24 hours for three days.

## RESULTS

### Differential expression analysis of m6A-related lncRNAs

**Figure 2:**
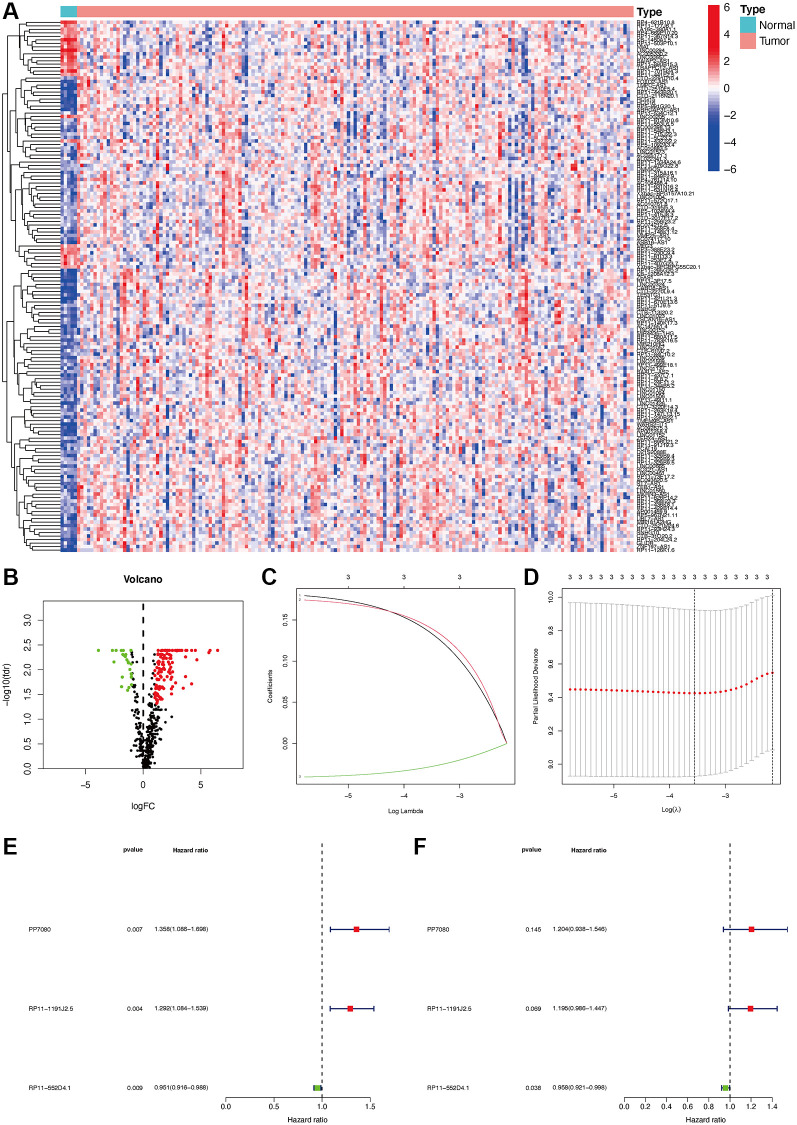
**Establishment of a risk assessment model based on DEmlncRNA differentially expressed m6A-related lncRNAs (DEmlncRNAs).** The heatmap (**A**) and a volcano plot (**B**) were displayed. Establishment of the LASSO regression (**C** and **D**). 3 DEmlncRNA were shown by a forest map with univariate and multivariate Cox analysis (**E** and **F**).

The gene transfer format (GTF) files from Ensemble were used to differentiate the noncoding and coding RNAs, and m6A-related lncRNAs were identified based on a co-expression form. In total, 460 m6A-related lncRNAs were identified, and among them, 152 were DEmlncRNAs. Among which, 127 were up-regulated and 25 were down-regulated (Figure 2A, 2B).

### DEmlncRNA screening and risk model construction

**Figure 3:**
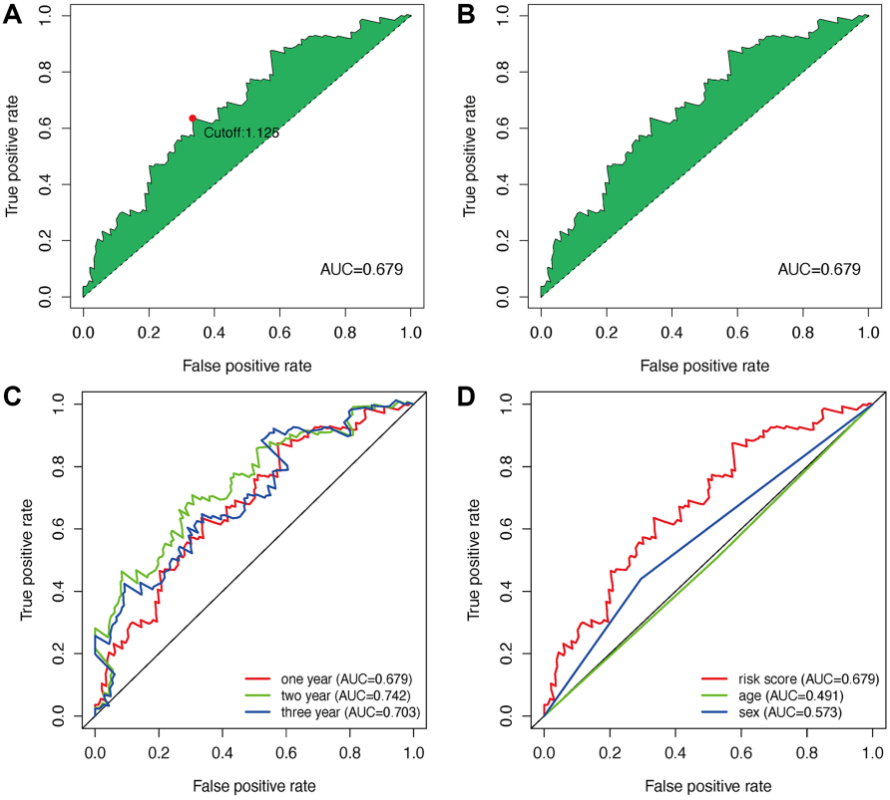
**Establishment of a risk assessment model based on DEmlncRNA.** The curve of each AUC value generated by ROCs of DEmlncRNAs was drawn, and the highest point of AUC was determined; The maximum inflection point is the cut-off point acquired from AIC (**A** and **B**). The 1-year, 2-year, and 3-year ROC of the optimal model showed that all AUC values exceeded 0.65 (**C**). Compared with other common clinical features, the 1-year ROC curves showed the superiority of the risk score against age and gender (**D**).

152 DEmlncRNAs were further identified and LASSO analysis was applied to find differentially-expressed lncRNAs. Figure 2C–2F shows the DElncRNAs. At the same time, the maximum AUC value was at 0.679, and the optimal DEmlncRNAs were identified (Figure 3A). We also calculated the Akaike information criterion (AIC) to look for the maximum inflection point as the cut-off point in the 3-year ROC curve (Figure 3B). ROC curves for one, two, and three years were drawn respectively, and all AUC values were more than 0.65 (Figure 3C). In addition, the one-year AUC value was much better against common clinical features. (Figure 3D).

### Risk model prediction in the clinical features

**Figure 4:**
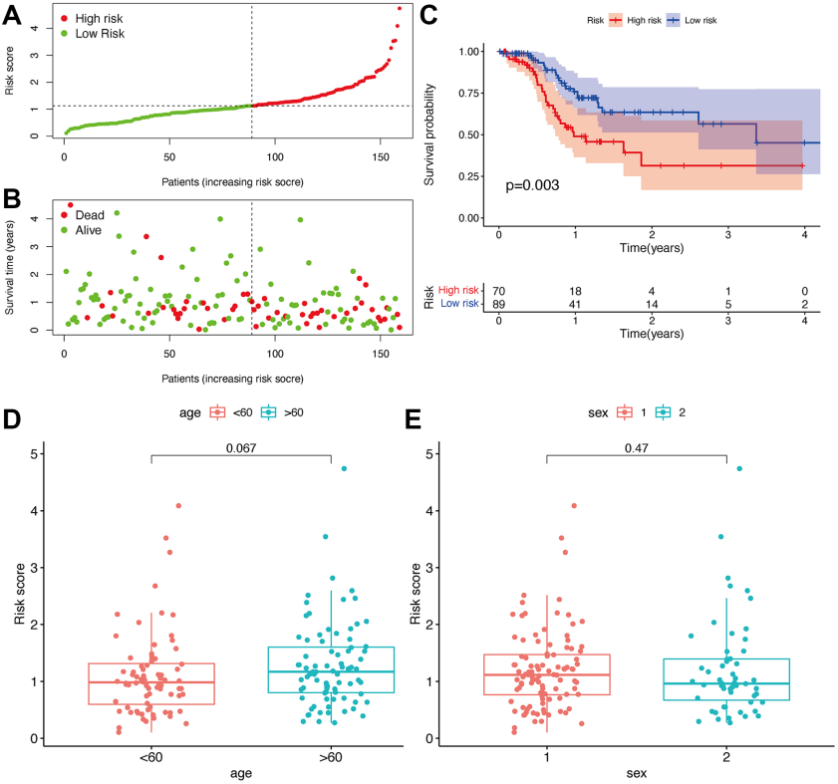
**Prognostic prediction of the risk assessment model.** Risk scores (**A**) and survival outcome (**B**) of each case. Kaplan-Meier survival curve of high-risk group and low-risk group (**C**). The scatter plot showed that age (**D**) and sex (**E**) were not significantly correlated with a risk score.

**Figure 5:**
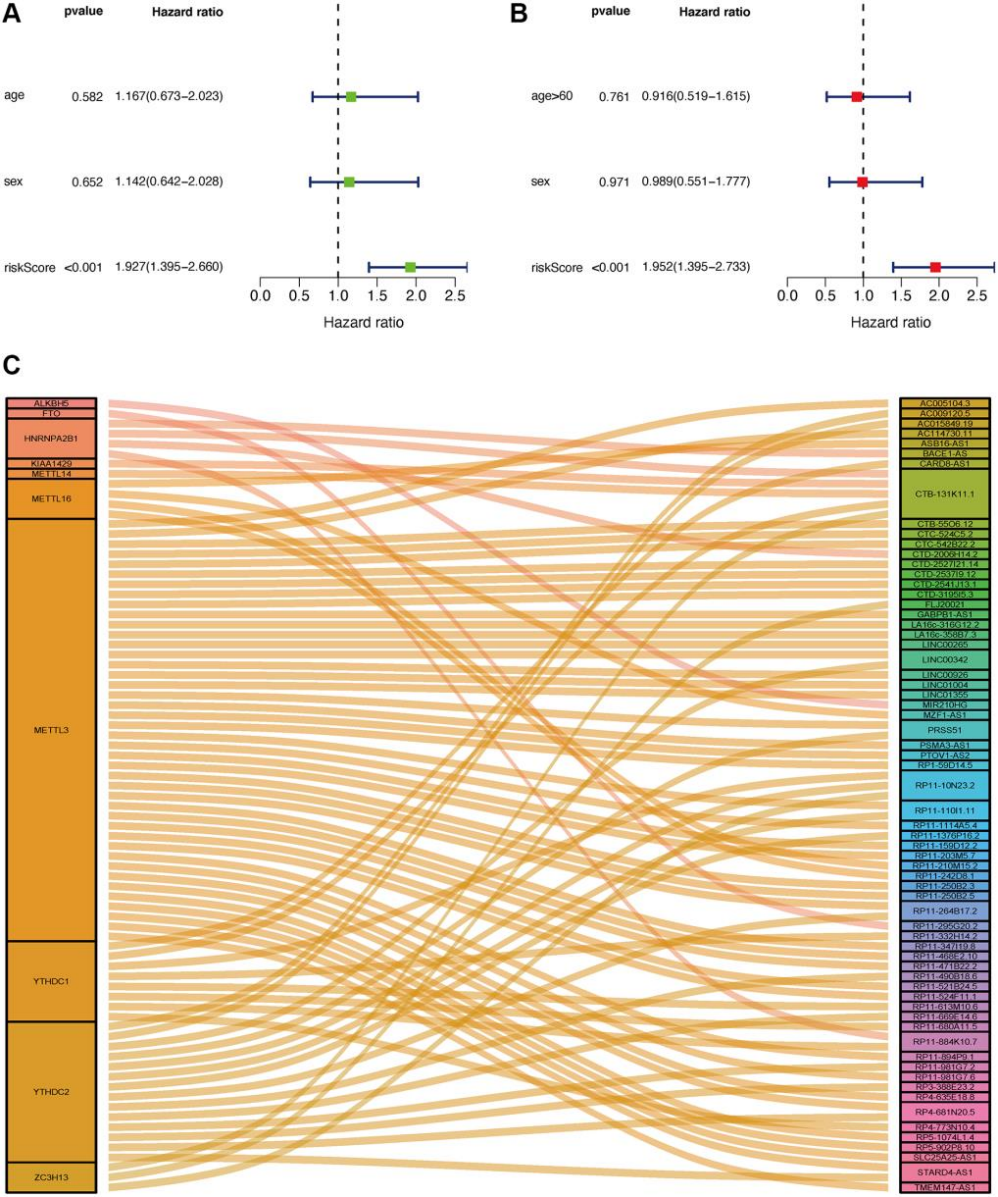
**Application of risk assessment model in the clinical evaluation.** Univariate and multivariate Cox regression analyses were carried out to analyze the clinicopathological features and shown by a forest map (**A** and **B**). Sankey Plot shows the link between m6A genes and related lncRNAs (**C**).

Seventy cases and 89 cases were classified into high- and low-risk groups based on the cut-off value. Figure 4A and 4B show the riskScores and survival rate of each patient. We found the outcome in patients from the high-risk group was poorer than that from the low-risk group, shown by KM analysis (*p* = 0.003) (Figure 4C). Then, a Chi-square test was applied to investigate the relationship between the risk model in GBM and clinical features. The scatter plots examined through Wilcoxon signed-rank test displayed age and gender (Figure 4D, 4E) were not significantly correlated with the risk model. Consequently, univariate and multiple Cox model showed that riskScores is an independent predicting factor (*p* < 0.001, HR = 1.927, 95% CI [1.395–2.660]) (Figure 5A), and riskScores (*p* < 0.001, HR = 1.952, 95% CI [1.395–2.733]) (Figure 5B). The m6A-lncRNA coexpression network was visualized using the Sankey diagram in Figure 5C, and 152 m6A-related lncRNAs were discerned as m6A-related lncRNAs.

### The risk model is correlated with immune status in GBM

**Figure 6:**
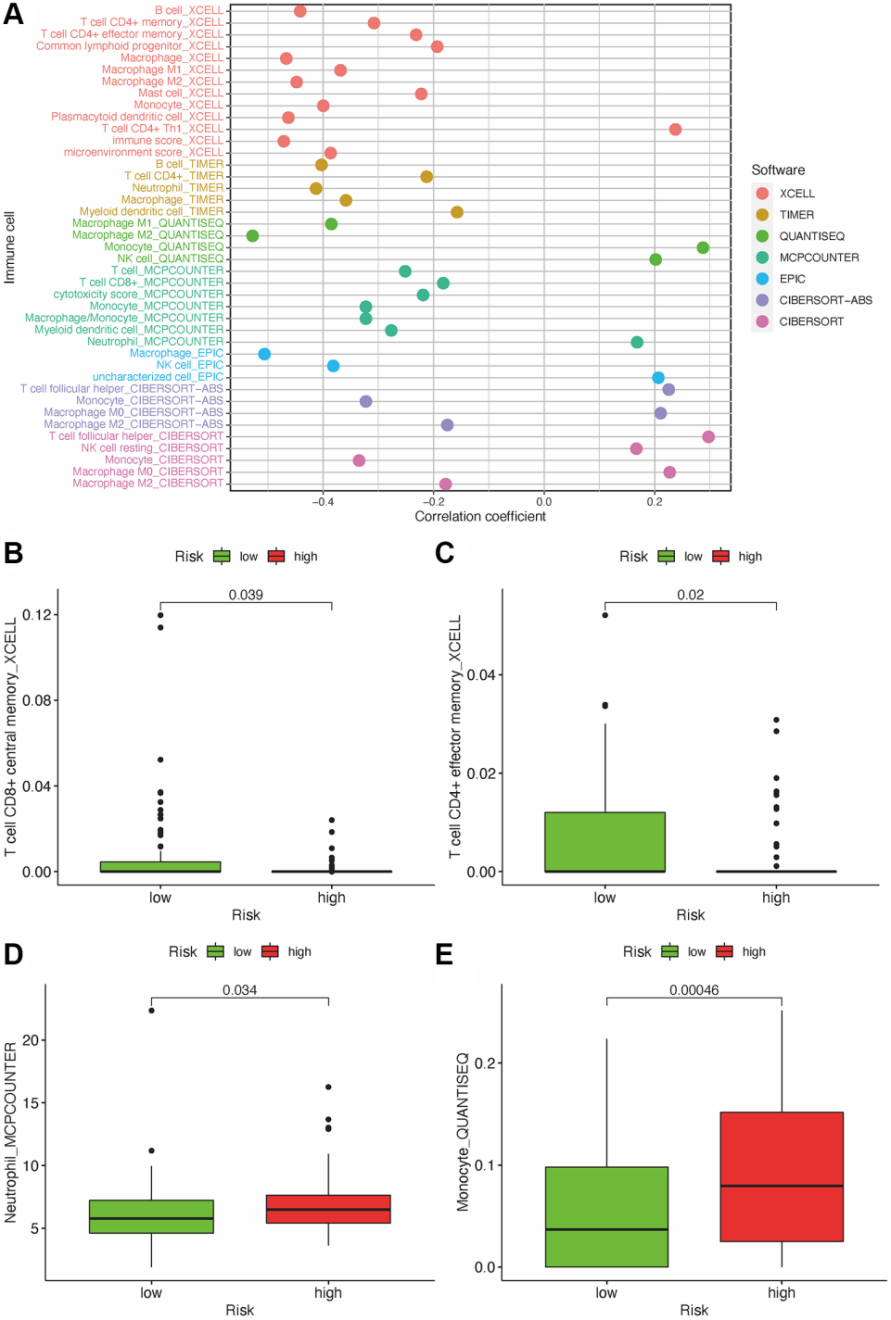
**Estimate tumor-infiltrating cells through risk assessment model.** (**A**) Association between the risk scores and TILs. High-risk group has higher neutrophils and monocytes and a lower proportion of CD4+ and CD8+ T cells (**B**–**E**).

**Figure 7:**
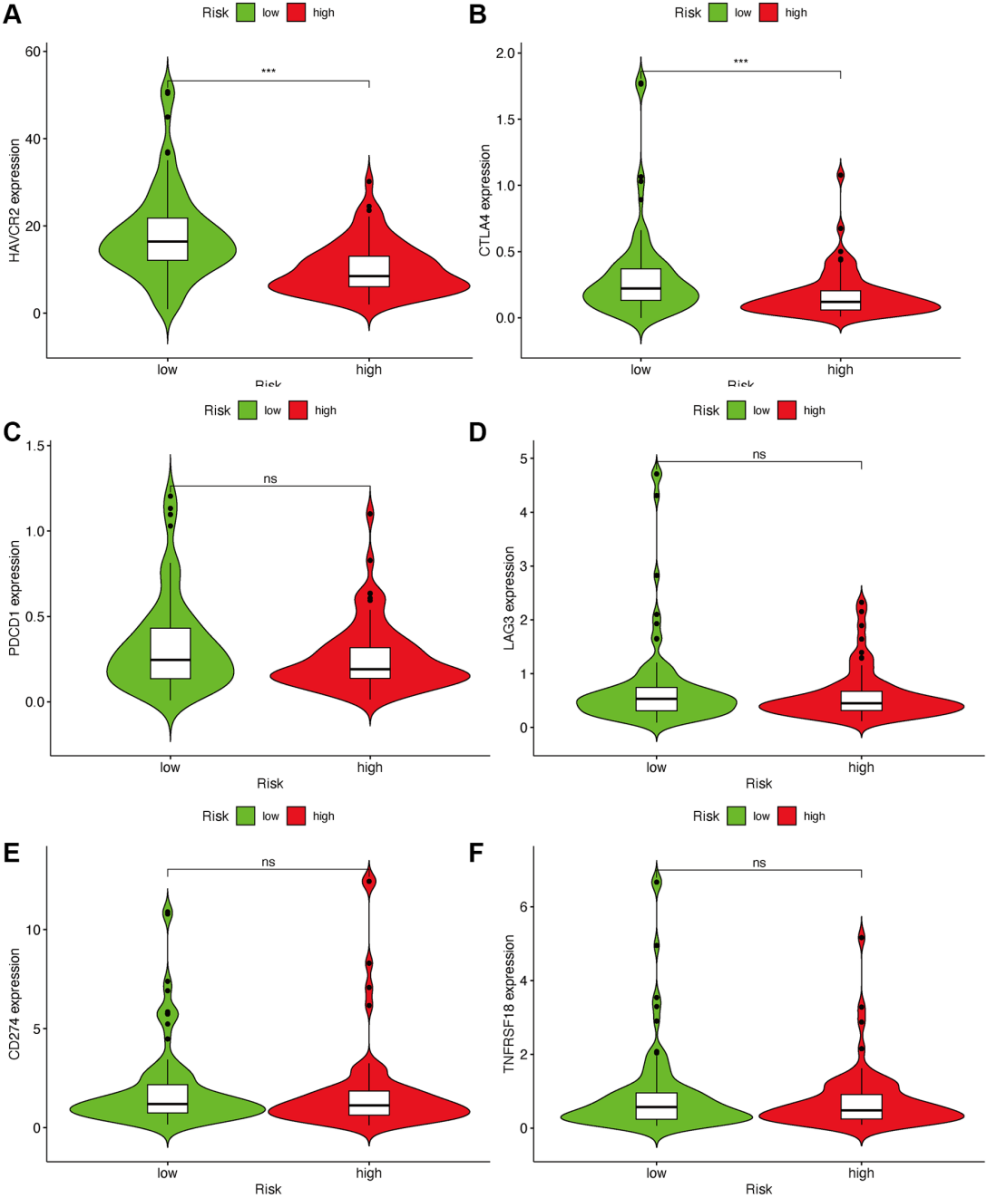
**Estimate immunosuppressive molecules through risk assessment model.** High-risk scores were associated with the expression of HAVCR2 (**A**) and CTLA4 (**B**). There was no statistical difference between the risk scores and immune-related genes, such as PDCD1 (**C**), LAG3 (**D**), CD274 (**E**) and TNFRSF18 (**F**).

As lncRNAs play a role in the immune infiltration of GBM, we next explored whether this model correlates with the immune infiltration status. Spearman correlation was performed, and the lollipop diagram showed their relationships (Figure 6A). We found that a positive correlation between the high-risk group and tumor-infiltrating immune cells, such as neutrophils and monocytes (Figure 6D, 6E), was negatively correlated with CD8+T cells and CD4+ cells (Figure 6B, 6C). As ICIs have an important role in treating solid tumors, however, their roles in GBM remain elusive, we further investigated the association between the risk model and ICI markers. Our results demonstrated that high-risk group was correlated with the expression level of HAVCR2 (*p* < 0.001) and CTLA4 (*p* < 0.001) (Figure 7A, 7B). There was no significant difference between the risk scores and ICI related-genes, such as LAG3, CD276, PDCD1, and TNFRSF18 (All *p* > 0.05, Figure 7C–7F).

### Correlational assessment of risk model and chemotherapy drugs

**Figure 8:**
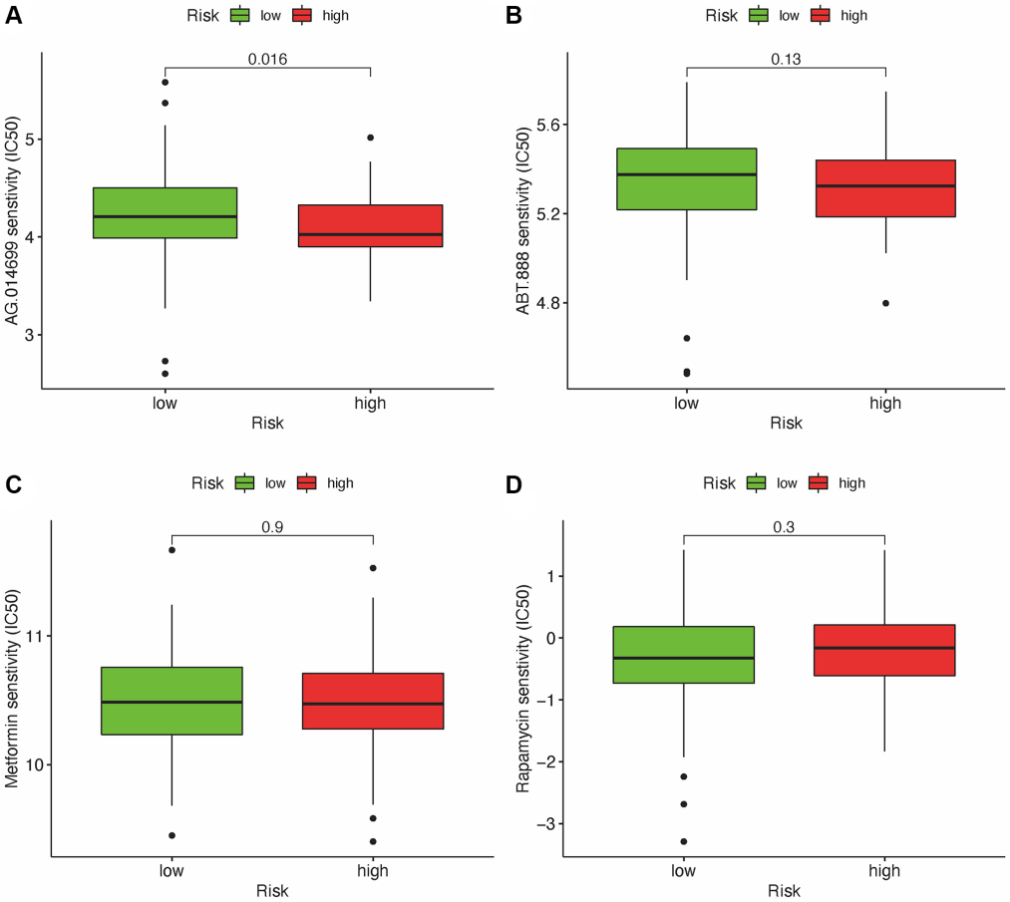
**Relationship between risk scores and IC50 of chemotherapeutics.** High-risk score was along with a lower IC50 for AG.014699 (*p* = 0.016): AG.l014599 (**A**), ABT.888 (**B**), Metformin (**C**) and Rapamycin (**D**).

Besides the relationship with ICIs, we further explored the relationship between the risk and efficacy of commonly used chemotherapy drugs for GBM. The results showed that the high-risk group had a higher half inhibitory concentration (IC50) of chemotherapy drugs for metformin (*p* < 0.05), while a lower IC50 for AG.014699, PARP inhibitor (*p* < 0.001), indicating that this risk model could be used as a potential predictor for the chemotherapy sensitivity (Figure 8A–8D).

### Validation of the independent risk gene RP11-552D4.1 in CGGA

Because the expression level of RP11-552D4.1 is an independent factor in the survival prediction for GBM (Figure 2F, with a *p*-value = 0.038), we next investigated its expression in Chinese Genome Atlas (CGGA), we first explored the expression of RP11-552D4.1 in different subtypes of CGGA and found it was lower in the Proneural group. The AUC of ROC in predicting RP11-552D4.1 for CGGA subtype (Proneural) is 0.637. In addition, the expression of RP11-552D4.1 is lower in IDH-1 mutant type compared to IDH-1 wildtype groups. As for the survival status, we found the expression of RP11-552D4.1 is positively associated with the survival status in WHO IV, while negatively correlated with the OS in WHO II, although it did not reach the statistical difference (*p* = 0.4 and 0.072, respectively). This might be due to the smaller sample size in CGGA compared to the TCGA dataset Supplementary Figure 1. Consistently, in the TCGA dataset, GBM patients with higher expression of RP11-552D4.1 has a longer survival period, while LGG patients with higher expression of this gene shows a shorter survival period (Supplementary Figures 2 and 3).

### RP11-552D4.1 facilitates neuronal survival

**Figure 9:**
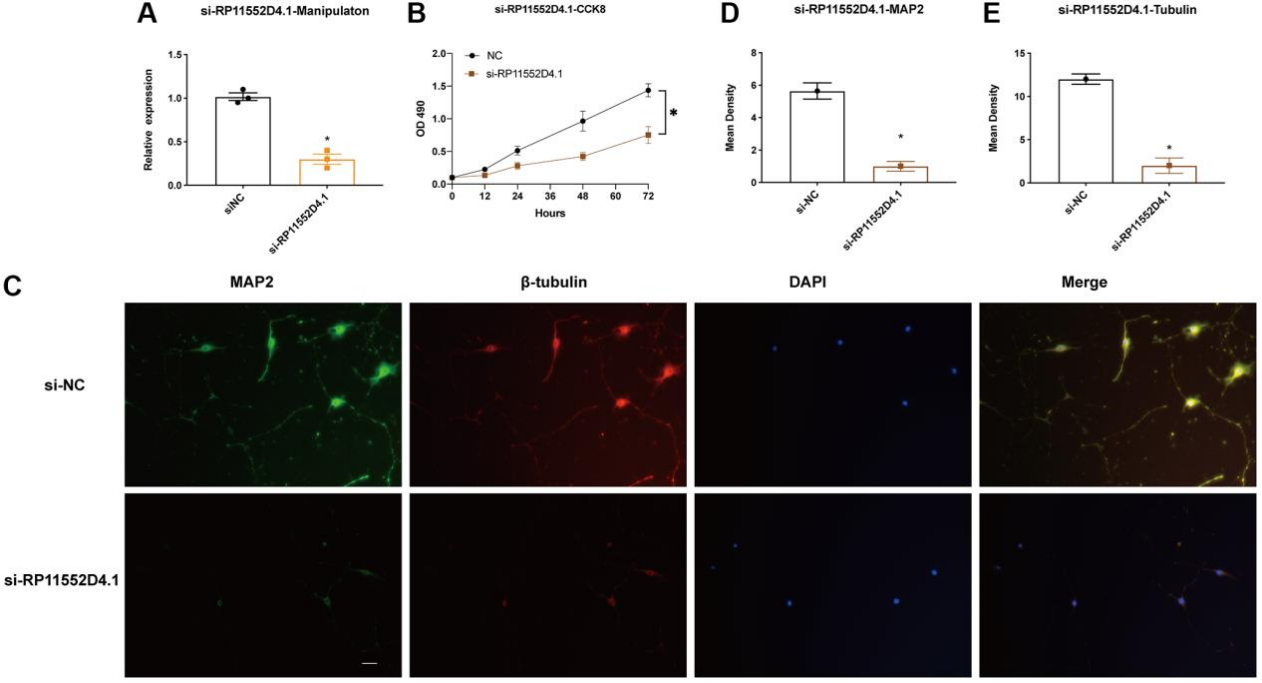
**RP11-552D4.1 facilitates neuronal proliferation.** (**A**) The expression of RP11-552D4.1 in si-RP11 was verified. (**B**) After primary cortical neurons were transfected with si-NC or si-RP11-552D4.1, a CCK-8 assay was applied to assess the cell proliferation. (**C**) Effect of si-RP11-552D4.1 on neuronal proliferation (Green: MAP2, Red: β-tubulin, Merged with DAPI). (**D, E**) Quantification of the mean density of MAP2 and β-tubulin. The scale bar is 100 μm. ^*^P < 0.05 (compared to the si-NC group).

**Figure 10:**
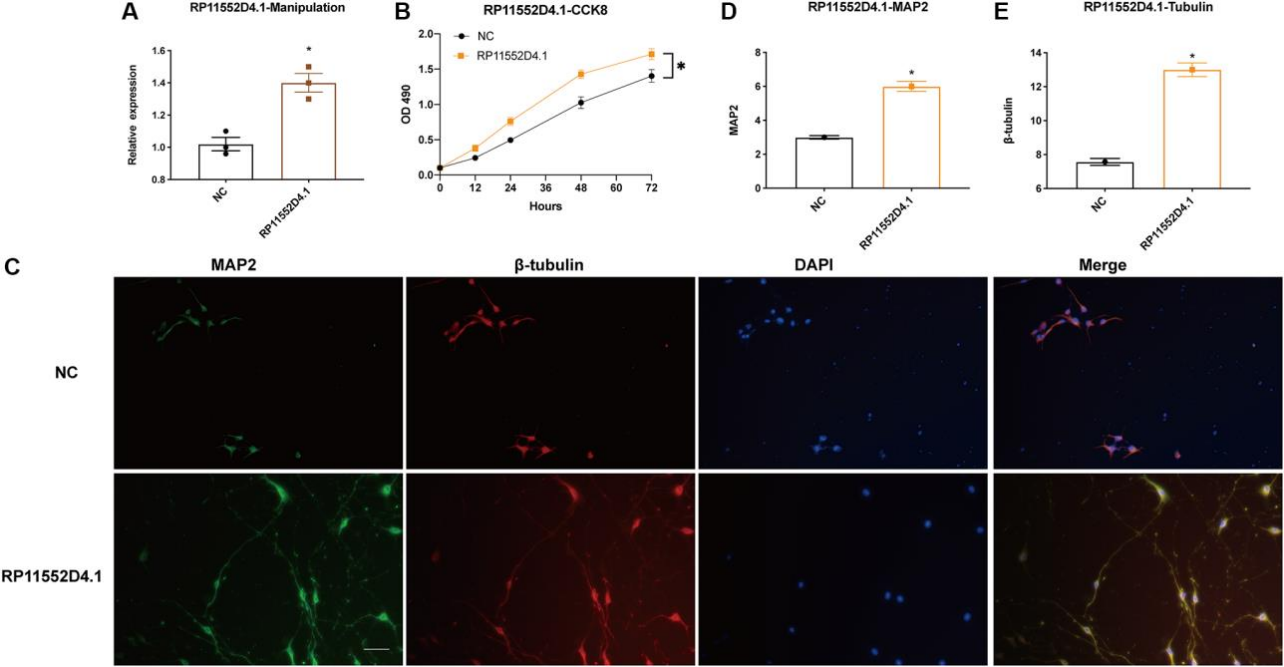
**Si-RP11-552D4.1 inhibits neuronal proliferation.** (**A**) Expression of RP11-552D4.1 in overexpressed RP11-552D4.1 was verified. (**B**) Primary cortical neurons were transfected with NC or RP11-552D4.1 plasmid. (**C**) Effect of RP11-552D4.1 on neuronal proliferation (Green: MAP2, Red: β-tubulin, Merged with DAPI). (**D**, **E**) Quantification of the mean density of MAP2 and β-tubulin. The scale bar is 100 μm. ^*^P < 0.05 (compared to the NC group).

From both TCGA and CGGA dataset, a similar trend between the expression of RP11-552D4.1 and survival outcome was identified, we next investigated the effect of RP11-552D4.1 on primary cultured neurons. Si- RP11-552D4.1 was used to knock down the expression of RP11-552D4.1 and si-NC was used as a control. CCK-8 assays indicated that primary cultured neurons transfected with si-RP11-552D4.1 showed decreased cell proliferation (Figure 9A and 9B). Similarly, transfection with si- RP11-552D4.1 also significantly reduced the expression of MAP2 and β-tubulin in cell culture (^*^P < 0.05, Figure 9C–9E). In contrast, overexpressed RP11-552D4.1 could upregulate the CCK level of primary neurons and increase the expression of MAP2 and β-tubulin as well (Figure 10).

## DISCUSSION

M6A-related lncRNA is a recent hot topic in the neuroscience field. Current studies have investigated the correlation between the m6A-related lncRNA and the risk of cancer. However, its role in glioma, even GBM is missing. So, whether the expression level of m6A-related lncRNAs has a relationship with the outcome of GBM patients’ needs to be addressed. The lncRNAs included in this model are related to m6A-genes based on co-expressed methods, so these lncRNAs may be regulated by m6A genes.

A recent review paper summarized m6A RNA methylation in brain tumor and reported that several m6A molecules differentially expressed in glioma from the CGGA dataset [23]. However, most m6A molecules directly regulated by miRNAs or indirectly adjusted by lncRNAs or circRNAs with a competitive endogenous RNA mechanism. There were very few reports regarding the lncRNAs directly modified by m6A methylation. Meanwhile, another bioinformatical study focusing on the m6A regulators in GBM identified that the risk score consists of HNRNPC, ALKBH5 and FTO could predict the survival outcome in GBM patients independently [24]. Again, this study did not further investigate the target genes of these m6A regulators. Therefore, we identified the prognosis-related DEmlncRNAs to construct a reliable model to predict the OS in GBM patients. Univariate and multivariate Cox models demonstrated that this risk model could predict the prognostic outcome of GBM patients, and it is independent of other clinical indices, such as patients’ age and gender. The risk score obtained from the model was more accurate against other clinical features in predicting survival status based on ROC analysis.

In addition, the chemotherapy sensitivity for GBM was investigated. The difference between risk groups and immune status and ICI genes were also investigated, which suggested this risk model was able to classify GBM patients who might be suitable for immunotherapy.

The response of ICI blockers is associated with TILs [25–27]. Our study applied customized immunol gene sets to study the correlation between risk factors and TILs. Our results demonstrated that DEmlncRNAs show a positive relationship with tumor-infiltrating immune cells such as neutrophils and monocytes, while negatively correlated with  CD4+ and CD8+T cells. In addition, our study indicates that the high-risk group in GBM was associated with chemotherapy sensitivity such as PARP inhibitor, instead of metformin, which provides potential targets for immunotherapy in GBM.

RP11-552D4.1 is previously found to decrease in heart failure [28], which indicates that it might exert its function on cellular proliferation. In our study, we investigated the effect of RP11-552D4.1 on the primary cultured neurons and found the knockdown of RP11-552D4.1 could inhibit the proliferation of neurons while overexpressed RP11-552D4.1 have the opposite effect, which indicates that RP11-552D4.1 is able to facilitate neuronal proliferation. And this is consistent with our findings that RP11-552D4.1 could become an independent risk factor for glioblastoma. From the Ensemble website, we could find the Ensembl ID of RP11-552D4.1 is ENSG00000228058 and the official symbol is LINC10736.

After literature review, LINC01736 is previously found to act as a protective factor in THCA, although with a high expression [29]. We, therefore, investigated the expression of related prognosis in TCGA and found the expression of LINC01736 is all increased in GBM, LGG, and THCA, however, it is positively correlated with the survival status in LGG, while negatively associated with the OS in THCA and GBM (the latter did not reach the statistical difference) from the KM plot (Supplementary Figure 2). Then, we investigated the LINC01736-related immune cell infiltration with the ssGSEA method [30]. We found LINC01736 is positively associated with the NK CD56bright enrichment in THCA and GBM (the latter did not reach the statistical difference), and negatively correlated with the NK CD56bright enrichment in LGG (Supplementary Figure 3), which suggests that LINC01736 exerts a different role in NK cell recruitment in different tumors. This explained why the expression of LINC01736 is increased in GBM and THCA, and seems to be a protectant factor in these tumors.

We have to commit that there are several limitations in this study. Firstly, the data was from public datasets and the sensitivity of drugs commonly used in the treatment of GBM, such as temozolomide has not been investigated; Secondly, the established risk model needs external verification, such as *in-vivo* study because the expression level of GBM shows heterogeneity, which results in the unreliability of the algorithm model. Although the lack of *in-vivo* validation, we applied COX and LASSO methods to establish the risk model with an independent prediction ability and identified one novel m6A-related lncRNA and further *in-vivo* studies are needed to validate its role in anti-GBM.

## CONCLUSION

In summary, we used m6A-related lncRNAs to construct a risk model that exactly predicts the outcomes in GBM patients. Furthermore, this model reflects the dysregulated immune infiltration status in GBM and provides potential targets for immunotherapy.

## Supplementary Materials

Supplementary Figures
