# Supplementary material for: RP11-552D4.1: a novel m6a-related LncRNA associated with immune status in glioblastoma
Source: Aging (Albany NY). 2022 Jul 18;14(18):7348–63. doi: 10.18632/aging.204177 (PMC9550243; doi:10.18632/aging.204177)
Supplement: Supplementary Figures [file aging-14-204177-s001.pdf]

SUPPLEMENTARY FIGURES

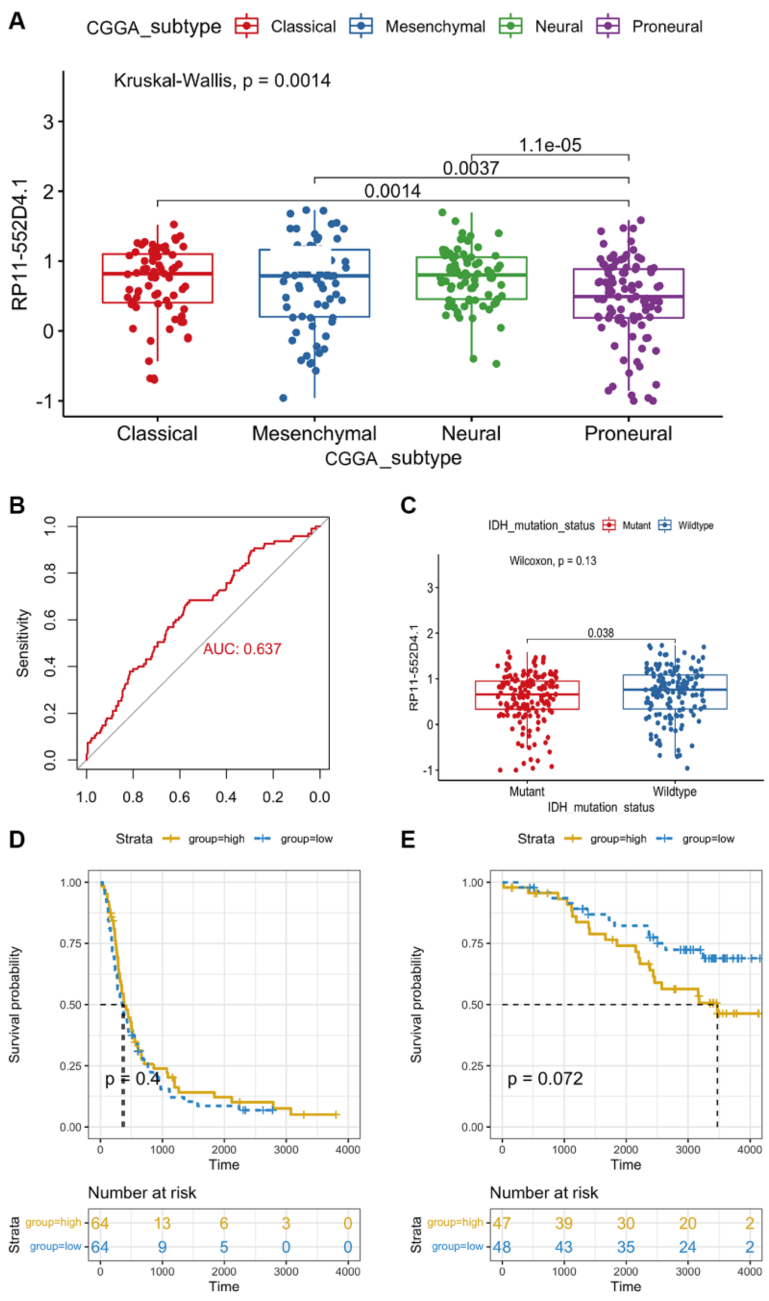

Supplementary Figure 1. The validation of RP11-552D4.1 in CGGA dataset. (**A**) The expression of RP11-552D4.1 in different subtypes of CGGA. (**B**) The prediction with ROC of RP11-552D4.1 for CGGA subtype (Proneural). (**C**) The different expression of RP11-552D4.1 between IDH-1 wt and IDH-1 mutation. (**D**, **E**) Its expression is negatively associated with the survival status in WHO IV, while positively correlated with the OS in LGG.

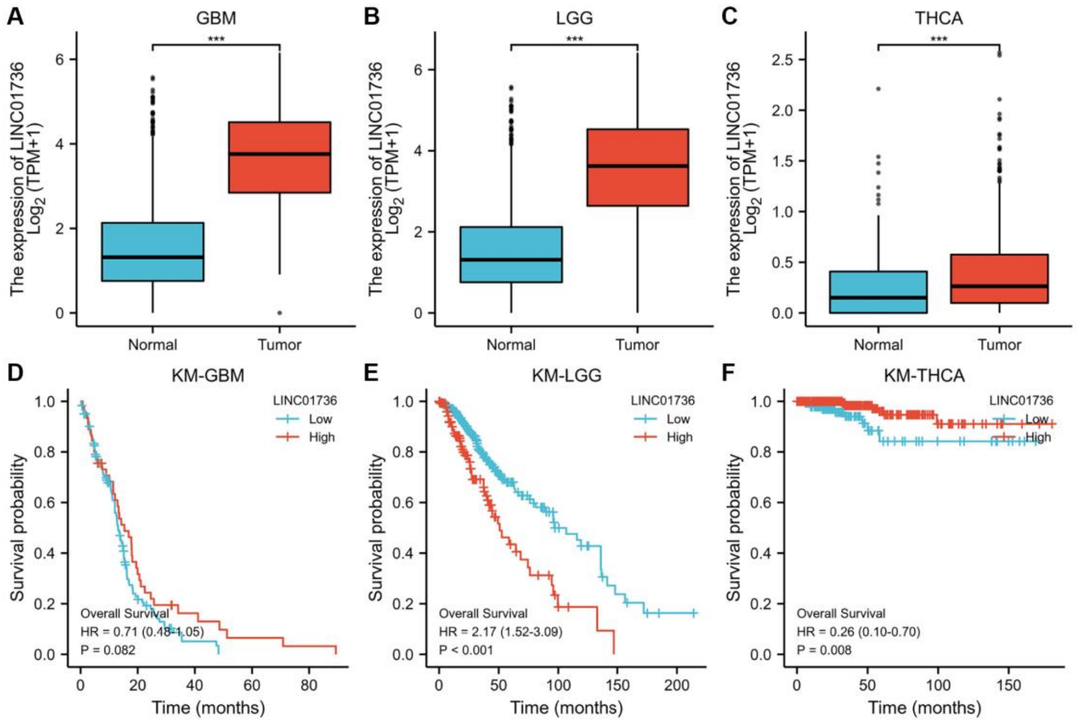

Supplementary Figure 2. The expression and prognosis of LINC01736 in GBM, LGG and THCA. (**A**–**C**) The expression level of LINC01736 is all increased in GBM, LGG and THCA. (**D**–**F**) Its expression is negatively associated with the survival status in GBM and THCA, while positively correlated with the OS in LGG.

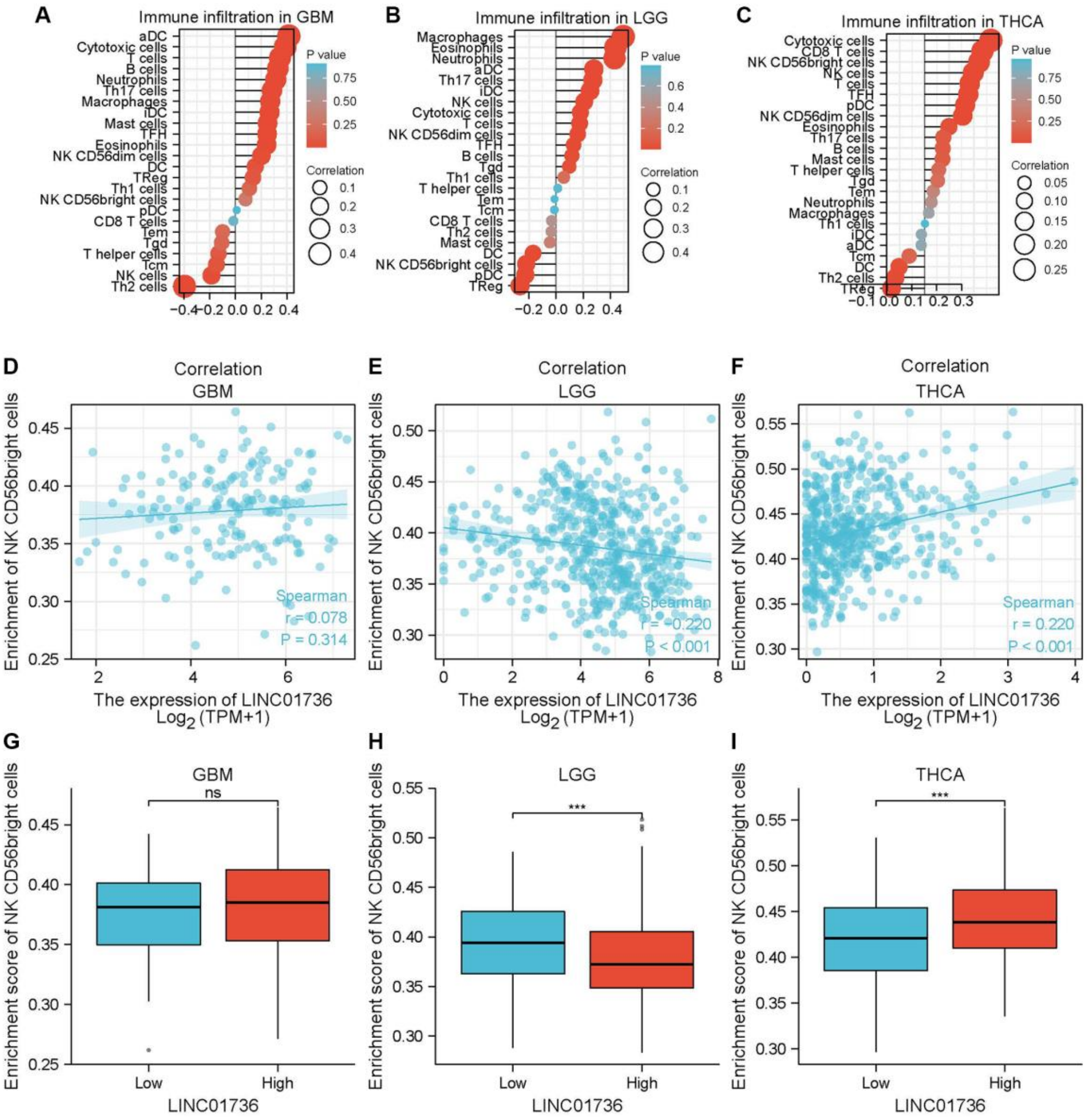

Supplementary Figure 3. The relationship between LINC01736 and the immune infiltration in GBM, LGG and THCA. (**A**–**C**) The lollipop plot shows the LINC01736-related immune cell infiltration in GBM, LGG and THCA; (**D**–**F**) The scatter plot of the correlation between LINC01736 and NK CD56 bright cells; (**G**–**I**) The bar graph of the expression of LINC01736 and NK CD56 bright cells.
